# A prospective cohort study on the pharmacokinetics of nivolumab in metastatic non-small cell lung cancer, melanoma, and renal cell cancer patients

**DOI:** 10.1186/s40425-019-0669-y

**Published:** 2019-07-19

**Authors:** Daan P. Hurkmans, Edwin A. Basak, Tanja van Dijk, Darlene Mercieca, Marco W. J. Schreurs, Annemarie J. M. Wijkhuijs, Sander Bins, Esther Oomen-de Hoop, Reno Debets, Markus Joerger, Arlette Odink, Astrid A. M. van der Veldt, Cor H. van der Leest, Joachim G. J. V. Aerts, Ron H. J. Mathijssen, Stijn L. W. Koolen

**Affiliations:** 1 Department of Medical Oncology, Erasmus MC Cancer Institute, Erasmus University Medical Center, PO Box 2040, 3000 CA Rotterdam, The Netherlands; 2 Department of Pulmonology, Erasmus MC Cancer Institute, Erasmus University Medical Center, PO Box 2040, 3000 CA Rotterdam, The Netherlands; 3 Department of Immunology, Erasmus University Medical Center, Rotterdam, The Netherlands; 4 Department of Medical Oncology and Hematology, Cantonal Hospital, St. Gallen, Switzerland; 5 Department of Radiology & Nuclear Medicine, Erasmus University Medical Center, Rotterdam, The Netherlands; 6 Department of Pulmonology, Amphia Hospital, Breda, The Netherlands; 7 Department of Hospital Pharmacy, Erasmus University Medical Center, Rotterdam, The Netherlands

**Keywords:** Nivolumab, PD-1, Pharmacokinetics, Solid tumors

## Abstract

**Background:**

Nivolumab is administered in a weight-based or fixed-flat dosing regimen. For patients with non-small cell lung cancer (NSCLC), a potential exposure-response relationship has recently been reported and may argue against the current dosing strategies. The primary objectives were to determine nivolumab pharmacokinetics (PK) and to assess the relationship between drug clearance and clinical outcome in NSCLC, melanoma, and renal cell cancer (RCC).

**Methods:**

In this prospective observational cohort study, individual estimates of nivolumab clearance and the impact of baseline covariates were determined using a population-PK model. Clearance was related to best overall response (RECISTv1.1), and stratified by tumor type.

**Results:**

Two-hundred-twenty-one patients with metastatic cancer receiving nivolumab-monotherapy were included of whom 1,715 plasma samples were analyzed. Three baseline parameters had a significant effect on drug clearance and were internally validated in the population-PK model: gender, BSA, and serum albumin. Women had 22% lower clearance compared to men, while the threshold of BSA and albumin that led to > 20% increase of clearance was > 2.2m^2^ and < 37.5 g/L, respectively. For NSCLC, drug clearance was 42% higher in patients with progressive disease (mean: 0.24; 95% CI: 0.22–0.27 L/day) compared to patients with partial/complete response (mean: 0.17; 95% CI: 0.15–0.19 L/day). A similar trend was observed in RCC, however, no clearance-response relationship was observed in melanoma.

**Conclusions:**

Based on the first real-world population-PK model of nivolumab, covariate analysis revealed a significant effect of gender, BSA, and albumin on nivolumab clearance. A clearance-response relationship was observed in NSCLC, with a non-significant trend in RCC, but not in melanoma. Individual pharmacology of nivolumab in NSCLC appears important and should be prospectively studied.

**Electronic supplementary material:**

The online version of this article (10.1186/s40425-019-0669-y) contains supplementary material, which is available to authorized users.

## Background

Nivolumab is a human immunoglobulin G4 (IgG4) monoclonal antibody (MoAb) that inhibits the interaction between the co-inhibitory immune receptor programmed death-1 (PD-1) and its ligands, PD-L1 and PD-L2. Nivolumab monotherapy has been approved for several indications, including advanced and metastatic melanoma [1], advanced clear-cell renal cell cancer (RCC), and metastatic non-small-cell lung cancer (NSCLC) [2, 3]. IgG4 MoAbs, such as nivolumab, are characterized by a relatively high molecular mass, leading to a slow distribution in tissues [4]. The elimination of nivolumab is very much alike endogenous immunoglobulins with a half-life of approximately 27 days [5] and a steady-state at 12 weeks.

In current clinical practice, nivolumab is administered in different schedules including 3 mg/kg Q2W, 240 mg flat dosing Q2W, and 480 mg flat dosing Q4W. The dosing of 3 mg/kg Q2W --approved by the Food and Drug Administration (FDA) in 2014 -- was based on dose-finding phase I/II studies, showing tolerability for the wide range of 0.1 to 10 mg/kg, and showing activity at 0.1 mg/kg Q2W and higher [6]. However, approval of nivolumab flat dosing (in March 2018), however, was solely based on in silico studies: selected flat doses were based on equivalence with initial dosing at median body weight of 80 kg. Population pharmacokinetic (PPK) modeling of data from approximately 100 clinical trials was used to simulate nivolumab concentrations and to compare flat dosing regimens (240 mg Q2W, 480 mg Q4W) with 3 mg/kg Q2W dosing [7, 8]. It is noteworthy that a previous model-based PPK analysis resulted in significant but not clinically relevant covariate effects, of which gender and body weight were the most important [9].

Few studies have assessed dose-response (D-R) and exposure-response (E-R) relationships of nivolumab. In a quantitative analysis [10] of a phase 1b dose-escalation study in 129 patients with NSCLC [6], a positive D-R relationship was found at 3 or 10 mg/kg versus 1 mg/kg. In addition, trough concentrations at steady state were correlated with objective response (OR) at 0.1 to 3 mg/kg in another cohort of patients with NSCLC [10]. A D-R relationship could not be demonstrated in patients with melanoma (*n* = 107) nor RCC (*n* = 34) at this dose range, but was only observed at 0.1 up to 1 mg/kg. In 221 melanoma patients treated in phase 1b [6] and 3 studies [11], absence of an E-R relationship was confirmed utilizing PPK modeling by relating the time-averaged nivolumab concentration to OR [12].

In a recent real-world study performed by our group, a steep positive E-R relationship of nivolumab was found for NSCLC (*n* = 76). Here, patients with a partial response (PR) had significant higher mean trough levels during therapy than patients with progressive disease (PD), and high exposure correlated significantly with better overall survival (OS) [13].

The present study addresses the PK of nivolumab in a real-world setting. The main objectives were 1) to define patient parameters influencing nivolumab pharmacokinetics and 2) to describe the relationship of systemic nivolumab clearance with objective response in patients with NSCLC, melanoma, and RCC. Secondary objectives include an exploratory analysis in regard to immune-related adverse events (irAEs), progression-free survival (PFS), and OS.

## Methods

### Patients and study design

Patients with advanced cancer who were treated with nivolumab between 20th April 2016 and 30th October 2018 at the Erasmus MC Cancer Institute (Rotterdam, The Netherlands) and the Amphia Hospital (Breda, The Netherlands) were included prospectively in this study (Dutch Trial Register number NTR7015/ NL6828), allowing for serial blood sampling during standard of care nivolumab treatment. The study was approved by the independent ethics committee (MEC 16–011) and all patients provided written informed consent. Blood samples were drawn prior to every 2-weekly nivolumab to measure trough concentrations. For those patients who gave extensive informed consent, intensive sampling was performed between the first and second administration of nivolumab. Patient characteristics and clinical data were prospectively collected.

### Pharmacokinetic measurements

For all patients (*n* = 221), nivolumab trough concentrations were determined for a selection of serum samples until end of treatment. Nivolumab serum concentrations were determined by an in-house developed and validated enzyme-linked immune sorbent assay (ELISA, as described previously [14]. Serum samples were selected to determine trough concentrations prior to each administration for the first 12 weeks, thereafter at evenly 12-weekly intervals until the end of treatment. For some patients (*n* = 3), intensive sampling allowed to determine nivolumab concentrations at 2 h, 2 days, and 1 week after the first administration in order to estimate a best-fit compartmental model.

### Data collection

The following baseline patient parameters were collected: gender, race, tumor type, performance status, age, body weight, body surface area (BSA), total volumetric tumor burden, serum creatinine, renal function, total serum protein, serum albumin, lactate dehydrogenase (LD) and leucocyte count. Performance status was determined according to Eastern Cooperative Oncology Group [15]. For NSCLC patients, weight loss was recorded and defined as a percentage of 2.5 or higher [16] during a period of 3 months prior to the first administration of nivolumab. BSA was calculated by the Mosteller equation [17]. Renal function was estimated using the Chronic Kidney Disease Epidemiology Collaboration (CKD-EPI) formula [18].

For a subgroup of NSCLC patients (*n* = 30), total volumetric tumor burden at baseline was assessed by a thoracic radiologist (A.O.) in a blinded manner using IntelliSpace Portal version 8 (Philips Medical Systems Nederland B.V., The Netherlands). Only primary tumor lesions with a long axis > 10 mm, lymph nodes with a short axis > 15 mm and metastatic lesions with a long axis > 10 mm were included. Total volumetric tumor burden was not assessed if the primary tumor was not identifiable or its boundaries could not be defined, e.g. due to surrounding atelectasis or radiation effects.

Best overall response (BOR) was assessed according to Response Evaluation Criteria in Solid Tumors version 1.1 (RECIST v1.1, [19]). A minimum duration of 90 days for stable disease (SD) was required. Confirmation of PR or complete response (CR) was not required. PFS was defined as the time from the first administration of nivolumab until PD or death due to any cause, whichever occurred first. OS was defined as the time from the first administration of nivolumab until death due to any cause. IrAEs were registered from start of treatment until end of follow-up according to National Cancer Institute Common Terminology Criteria for Adverse Events version 4.03 (NCI-CTCAE v4.03). Data cut-off for these analyses was set at 1st of January 2019.

### Pharmacokinetic modeling

To determine patient parameters influencing nivolumab PK (primary objective 1), nonlinear mixed effect modeling software, NONMEM (version 7.4; ICON, Development Solutions, MD) was used to analyze the PK data. The first-order conditional estimation method with interaction was used for parameter estimation. Pirana software version 2.9.7 (Pirana, www.pirana-software.com) was used as a modeling environment, and data were further handled in the latest R desktop version 1.1.453 (R-project, www.rproject.org).

A two-compartment PPK model was developed to best fit the nivolumab pharmacokinetics with individual estimates of systemic drug clearance (schematically shown in Additional file 1: Figure S1). Two-compartment PPK models have previously been described to best fit pharmacokinetics of monoclonal antibodies in blood [20]. Since we had only trough PK levels available, modelling of nivolumab distribution was challenging. Hence, we assumed that the central volume (V1) equals the peripheral volume of distribution (V2) as previously described for nivolumab [21].

Between-subject variability (BSV) was tested for clearance and distribution volume. The inclusion of BSV was evaluated according to the change of objective function value (OFV, *P* < 0.05) and shrinkage. A shrinkage value below 25% was considered acceptable [22].

BSV was modelled according to Eq. 1:

1 $$ {P}_i=P\bullet \mathit{\exp}\left({\eta}_i\right) $$

where P_i_ represents the parameter estimate for each individual patient (*i*), P represents the typical population parameter estimate and η_i_ represents BSV distributed according to *N* (0, ω^2^).

Residual errors were described by a proportional error model (Eq. 2):

2 $$ {C}_{obs, ij}={C}_{pred, ij}\times \left(1+{\varepsilon}_{p, ij}\right) $$

where C_obs,ij_ and C_pred,ij_ represent the observed and predicted concentration for the (*i)* th subject and the *(j)* th measurement, respectively. ε_p,ij_ represents the proportional error distributed according to *N* (0,σ^2^).

Covariates were added to the PPK model (initial model M_i_) to obtain a final model (final model M_f_). Potential covariates were selected based on clinical plausibility and tested by a stepwise approach with forward inclusion (threshold *p* < 0.01) and backward elimination (threshold *p* < 0.005, [23–25]). The covariates were tested on clearance (CL) by multiplying a typical clearance value (CL_TV_) with a factor for categorical (Factor_cat_) and continuous (Factor_con_) covariates (Eq. 3).

3 $$ CL={CL}_{TV}\times {Factor}_{cat}\times {Factor}_{con} $$

Categorical covariates were scored as ‘0’ or ‘1’. Equation 4 was applied for patients who scored ‘1’ in which *θ*_*x*_ represents the covariate effect size estimate. Continuous variables were tested with the PK model using Eq. 5 where *cov* represents the covariate measure, *cov*_*median*_ the population median of the covariate, and *θ*_*y*_ the covariate effect measure.

4 $$ {Factor}_{cat}=1+{\theta}_x $$

5 $$ {Factor}_{con}={\left(\frac{\mathit{\operatorname{cov}}}{{\mathit{\operatorname{cov}}}_{median}}\right)}^{\theta_y} $$

### Data analysis

Descriptive statistics included frequency and the median with range and inter-quartile range (IQR) of covariates. To analyze the relationship of systemic nivolumab clearance, which is inversely proportional to drug exposure, with treatment outcome (primary objective 2), patients were stratified by tumor type (NSCLC, melanoma, and RCC) and ranked according to BOR. To avoid potential confounding from covariates that may correlate with response, the initial model M_i_ was used to compare individual drug clearance estimates between different BOR groups (PD, SD, PR/CR). Equal variances among groups were assessed with Levene’s test, normal distribution was assessed using the skewness and kurtosis. Comparison of individual drug clearance was assessed for the three BOR groups by ANOVA and post-hoc independent samples t-tests.

To investigate the relationship of systemic nivolumab clearance with toxicity, patients were stratified by tumor type, grouped based on the occurrence of grade 0–2 or grade > 3 irAEs, and analyzed by independent samples t-test. To relate systemic nivolumab clearance to PFS and OS, NSCLC patients were grouped into quartiles: patients with low clearance (Q1) were compared with patients with high clearance (Q4) by the Kaplan-Meier approach. The relative risk of death or death/progression was assessed by the Cox proportional hazards model. Additional patient characteristics were included grouped by clearance quartile (Q1-Q4). A two-sided *p*-value < 0.05 was considered significant.

Post-processing of NONMEM generated data and statistical analysis was conducted with R and IBM SPSS Statistics version 24.0.0.1 (Chicago, IL).

## Results

**Fig. 1:**
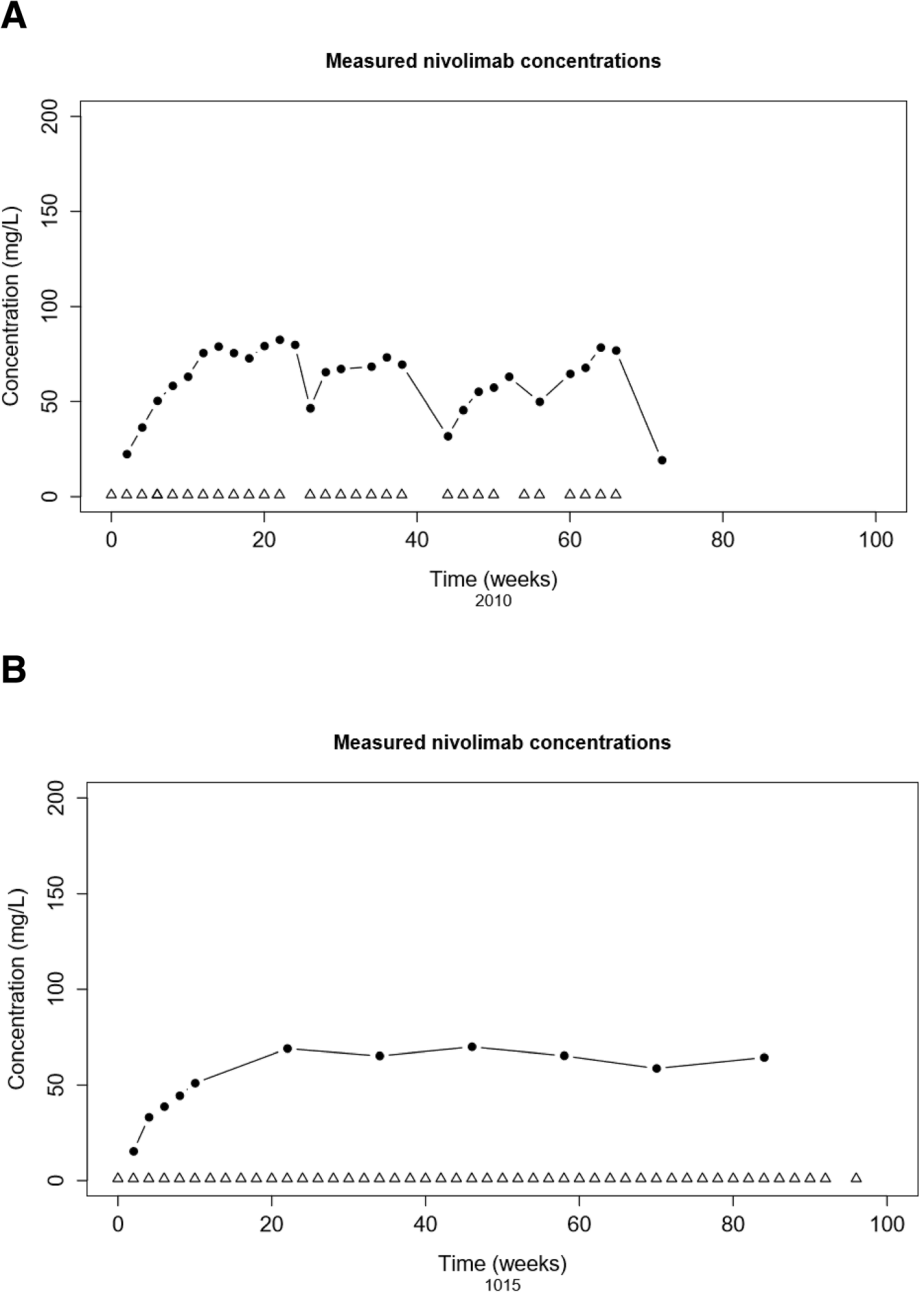
Patient examples. Example of two subjects (2010: NSCLC, 1015: melanoma patient) showing concentrations of nivolumab (mg/L) versus time (weeks), with received administrations of nivolumab being marked as open triangles. Single measurements are represented by closed circles. **a** Note that patient 2010 experienced several dose delays followed by a decrease of nivolumab concentrations that was in line with the approximate half-life time of 25 days, whereas **b** patient 2015 has had no dose delays and demonstrated a time to steady state concentrations of approximately 20 weeks

A total of 221 nivolumab-treated cancer patients were included in the PPK model (M_i_ and M_f_): NSCLC (71.4%), melanoma (21.7%), RCC (6.3%), and one mesothelioma patient. The patient characteristics are shown in Table 1. One patient received ipilimumab after initial treatment with nivolumab monotherapy, and was excluded from clearance-response, clearance-survival and clearance-toxicity analysis. Dosing was based on body weight (3 mg/kg Q2W), with an average dose of 240 mg per administration (IQR: 200–280 mg). The average number of nivolumab cycles administered per patient was 12. The overall median follow up time (from first administration of nivolumab to censoring) was 338 days (IQR: 145–487 days). A total of 1,715 measurements were available for PK analysis (average of 8 measurements per patient). Examples of nivolumab measurements and administrations over time from two patients, one with and one without dose delays, are shown in Fig. 1.

**Table 1:** Patient characteristics

Demographic Covariates Categorical		n (%)
Tumor Type		
NSCLC all types		158 (71.5)
Non-Squamous		96
Squamous		42
Unknown NSCLC type		20
Melanoma		48 (21.7)
RCC		14 (6.3)
Mesothelioma		1 (0.5)
Treatment		
Nivolumab monotherapy (3 mg/kg Q2W)		221 (100)
Gender		
Male		138 (62.4)
Female		83 (37.6)
Race		
Caucasian		195 (88.2)
Other		5 (2.3)
Unknown		21 (9.5)
WHO Performance Status		
0		63 (28.5)
1		103 (46.6)
2		4 (1.8)
Unknown		51 (23.1)
Weight loss prior to start therapy (only in NSCLC)		
Yes		36 (16.3)
No		81 (36.7)
Unknown		104 (47.1)
Demographic and Laboratory Covariates Continuous	Median (IQR)	n (%)
Age (yr)	65 (59–71)	221 (100)
Body Weight (kg)	78.5 (70–88)	220 (99.5)
Body Surface Area (m^2^)	1.95 (1.81–2.09)	205 (93)
Tumor Burden 3D (cm^3^; only in NSCLC)	18.6 (66–98)	25 (11)
Creatinine (μmol/L)	81 (66–98)	203 (92)
CKD (mL/min)	81 (62–90)	203 (92)
Total Protein (g/L)	73 (69–90)	163 (74)
Albumin (g/L)	42 (42–45)	174 (79)
LD (U/L)	215 (183–275)	196 (89)
Leucocytes (10^9^ cells/L)	7.7 (6.3–10.2)	203 (92)

Baseline covariates of patients

Abbreviations: *n* number of patients, *IQR* inter-quartile range, *CKD* CKD-EPI renal clearance, *LD* lactate dehydrogenase

### Gender, BSA and albumin influence nivolumab pharmacokinetics

**Fig. 2:**
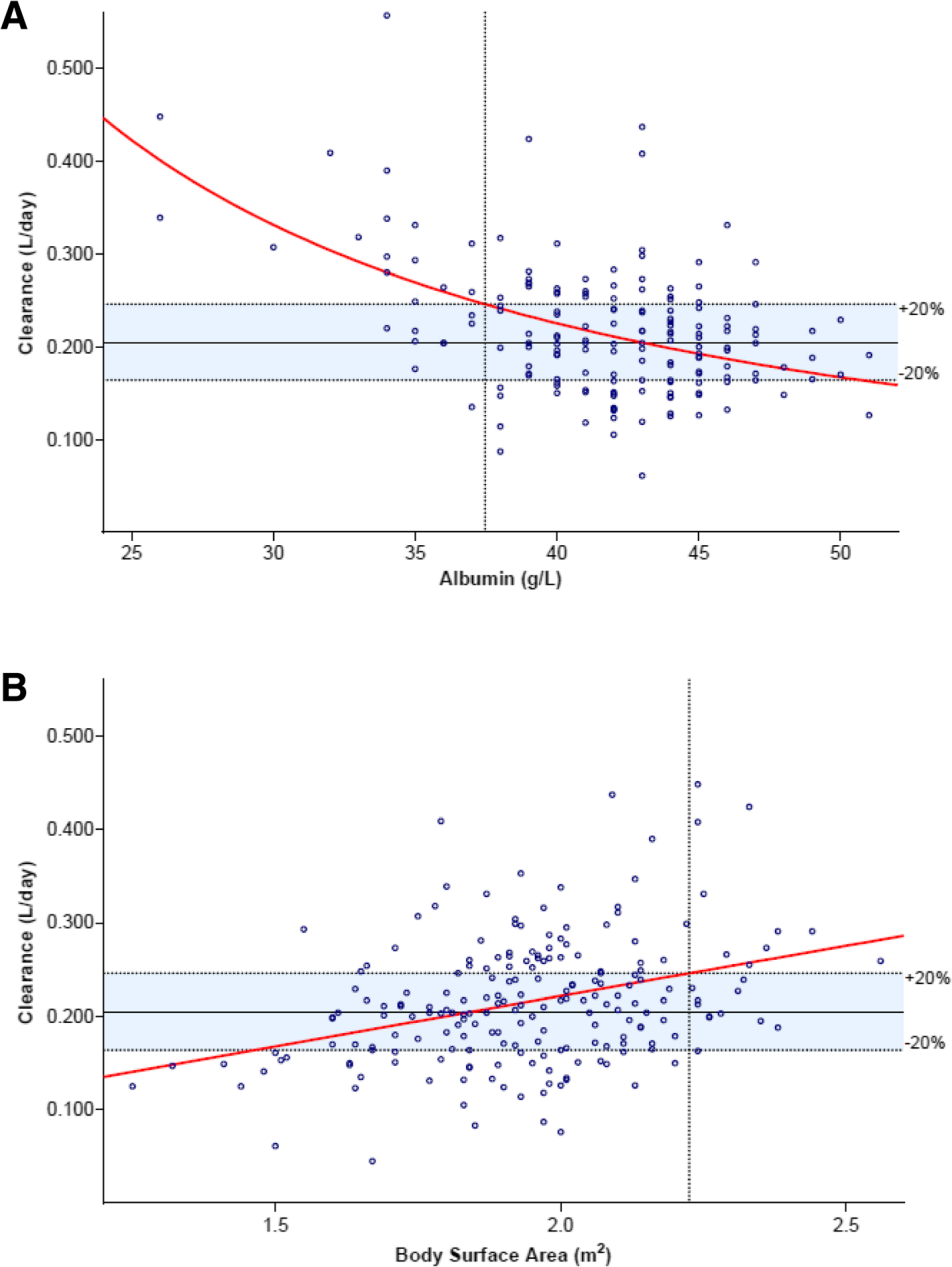
Parameter effect on clearance: **a** Estimated nivolumab clearance (L/day) as a function of **a** baseline serum albumin (g/L) and **b** body surface area (BSA; m^2^). Single measurements are represented by open circles. The red line predicts clearance according to the final PPK model (M_f_). The horizontal dotted lines marks the 20% increase of clearance, taking the mean clearance as reference (solid line). The vertical dotted line mark the threshold where nivolumab clearance is expected to be increased by > 20%

Continuous and categorical clinical covariates were incorporated in the final two-compartment model by forward inclusion and backward elimination (Additional file 1: Table S1), resulting in four covariates reaching the significance threshold, namely: gender, BSA, albumin, and body weight. BSA had a higher impact on nivolumab pharmacokinetics than weight; the latter being rejected by the backward elimination step. The parameter estimates according to the M_f_ are shown in Table 2 including the results of internal validation. The NONMEM model can be found Additional file 1: Appendix 1. Inter-individual variance was reduced from 37% (as indicated by M_i_) to 30.7% by incorporating these three covariates. Women had 22% lower clearance than men, as evidenced by a mean clearance of 0.185 and 0.237 L/day, respectively. The thresholds of BSA and baseline serum albumin that led to an estimated > 20% increase of systemic nivolumab clearance were > 2.2 m^2^ (BSA) and < 37.5 g/L (albumin), respectively (Fig. 2).

**Table 2:** Parameter estimates

Parameters	Units	Estimate	RSE (%)	Bootstrap estimate	Bootstrap 95% CI
Population parameters					
Clearance (CL)	L/day	0.211	3.5	0.211	0.196 to 0.226
Central volume of distribution (V1)	L	3.46	5.8	3.46	3.09 to 3.83
Peripheral volume of distribution (V2)	L	3.46	5.8	3.46	3.09 to 3.84
Inter-compartmental clearance (Q)	L/day	0.48	< 0.1	0.48	0.48 to 0.48
Covariate effects					
Female gender on CL	–	−0.17	29.1	−0.17	−0.27 to −0.06
BSA effect on CL	–	0.97	24.1	0.96	0.48 to 1.45
Albumin effect on CL	–	−1.34	19.8	−1.33	−1.83 to − 0.86
Between-subject variability					
Clearance (CL)	CV%	30.7	9	30.3	24.8 to 35.6
Residual unexplained variability					
Proportional error	CV%	31.8	8	31.8	29.1 to 34.2

Population parameters, covariate effects and between-subject variability according to the final population pharmacokinetic model (Mf). Abbreviations: *CL* clearance, *RSE* relative standard error, *CV%* percentage coefficient of variation, *CI* confidence interval. The shrinkage of the between-subject variability of clearance and the proportional error was 9.2 and 4%, respectively

### Correlation between drug clearance and clinical outcome

**Fig. 3:**
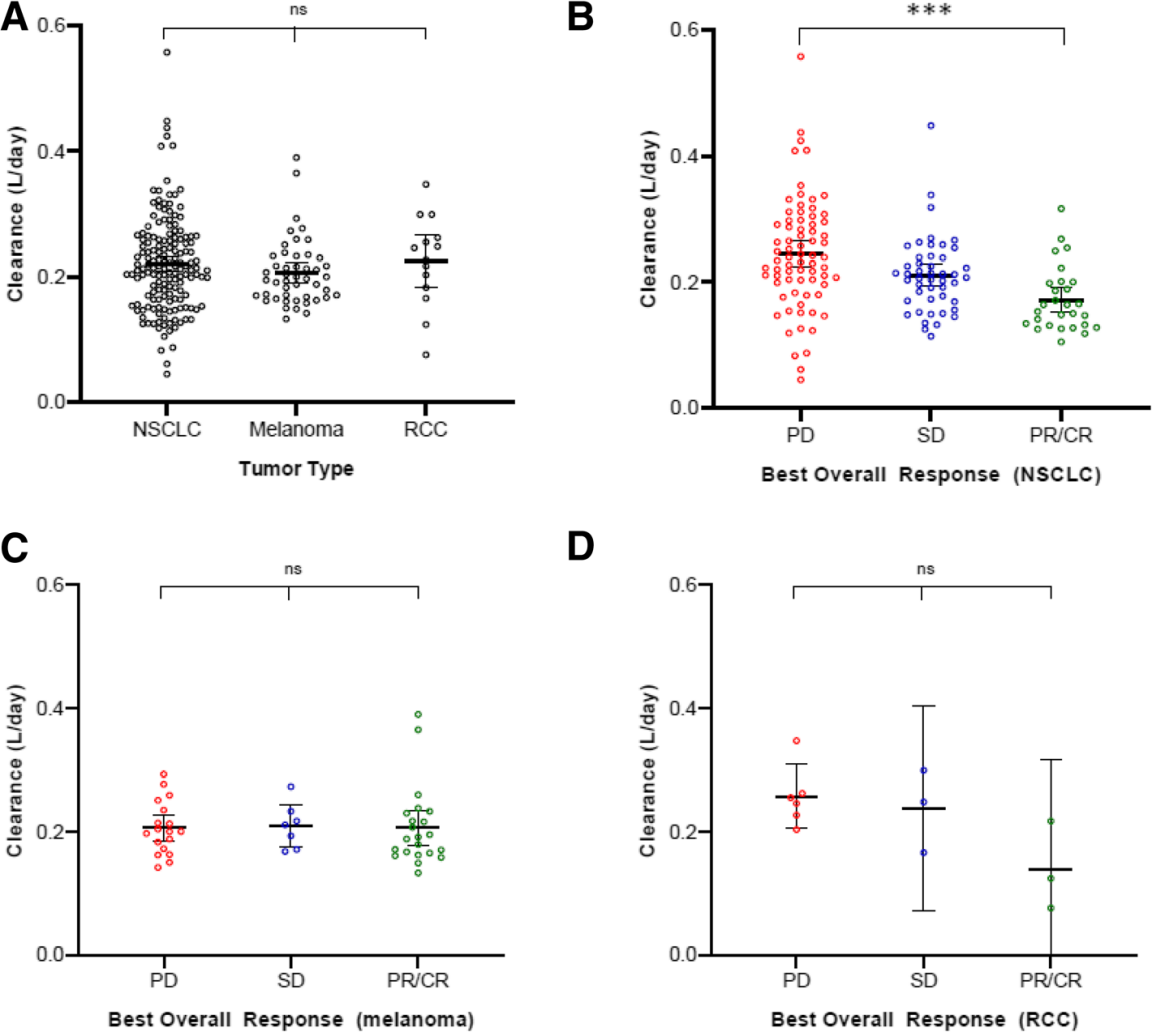
Clearance-response analysis: **a** Nivolumab clearance (L/day) of **a** all patients receiving nivolumab monotherapy grouped by best overall response (BOR), and stratified by **b** NSCLC, **c** melanoma, and **d** RCC. Single measurements are represented by open circles. Bars indicate the 95% confidence interval of the mean. Abbreviations: progressive disease (PD), stable disease (SD) and partial response/ complete response (PR/CR). *P*-values indicated by *** < 0.001 (post-hoc independent samples t-test)

Clinical outcome and occurrence of toxicity are shown in Additional file 1: Table S2. The initial model (M_i_) was used to investigate the relationship between individual clearance of nivolumab and clinical response or toxicity in NSCLC, melanoma, and RCC (Fig. 3b-d). A negative clearance-response relationship was found in patients with NSCLC (*p* < 0.001), as a significantly higher clearance of 41.8% was observed in patients with PD (mean: 0.244; 95% CI: 0.223–0.265 L/day) compared to patients with PR/CR (0.172; 0.152–0.192). Patients with SD were identified as an intermediate group (0.211; 0.193–0.228). A non-significant trend similarly to NSCLC was observed in RCC (*p* = 0.054). Of note, no clearance-response relationship was observed in melanoma (*p* = 0.987). A clearance-irAE relationship was not found for NSCLC, melanoma, or RCC (respectively *p* = 0.28, *p* = 0.84 and *p* = 0.92; Additional file 1: Figure S2B-D), nor for all three tumor types pooled together (*p* = 0.31; Additional file 1: Figure S2A).

**Fig. 4:**
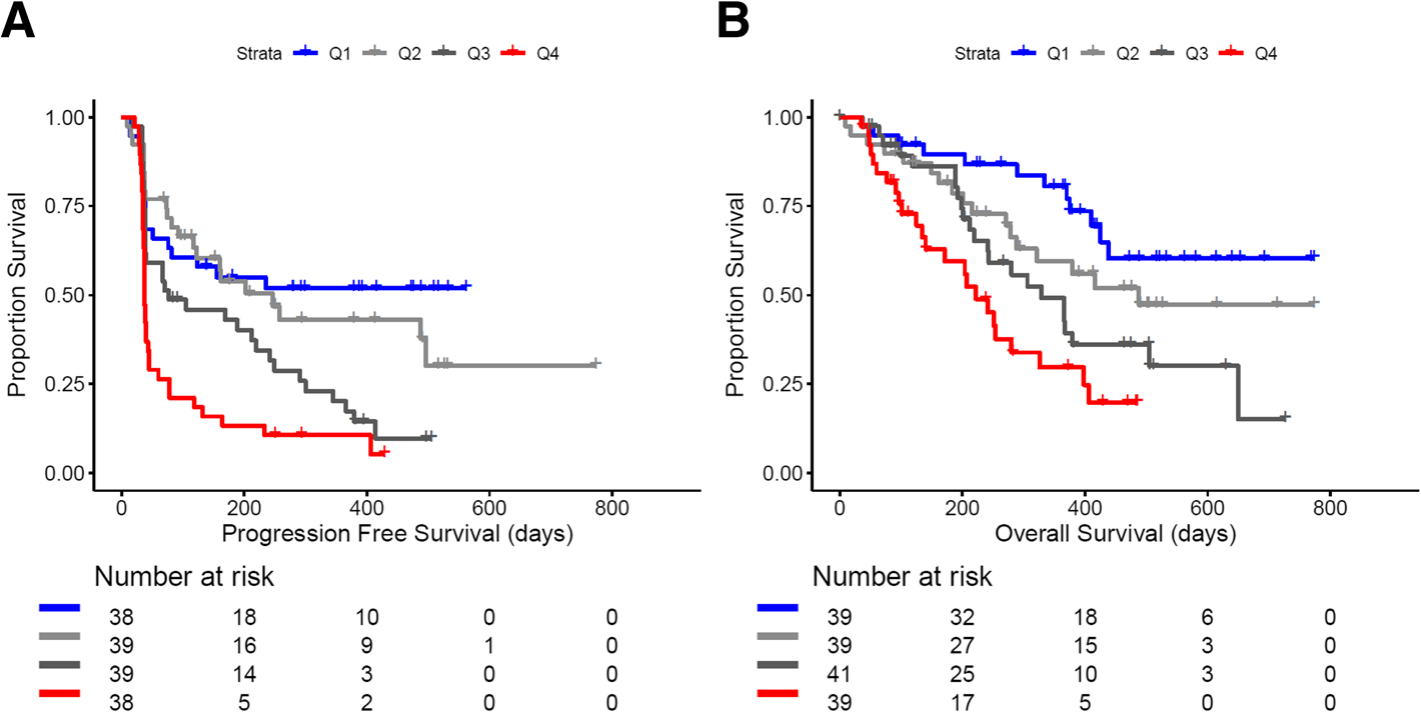
Kaplan-Meier curves: **a** progression-free survival (PFS) and **b** overall survival (OS) of NSCLC patients receiving nivolumab monotherapy stratified by clearance into 4 quartiles of clearance displayed by Kaplan-Meier methodology: Q1 [first quartile (blue); lowest clearance] - Q4 [fourth quartile (red); highest clearance]

There was no significant difference in drug clearance between tumor types (*p* = 0.47; Fig. 3a), corresponding with above-mentioned PPK modeling where tumor type as a categorical covariate did not reach the significance threshold. Notably, when patients with NSCLC were grouped by clearance, the lowest quartile of clearance was significantly associated with better PFS (HR 0.32; 95% CI: 0.18–0.57, *p* < 0.001) and OS (HR: 0.25; 95% CI: 0.12–0.51, *p* < 0.001) compared to patients with the highest quartile of clearance (Fig. 4a-b). Additionally, the patient characteristics grouped by quartile of clearance are shown in Additional file 1: Table S3.

## Discussion

In the current study, we showed that gender, baseline BSA, and serum albumin had a significant effect on the systemic clearance of nivolumab in the two compartment PPK model. These three covariates partially explained the high inter-individual variance (~ 37%) of drug clearance. Secondly, we have demonstrated the relationship between nivolumab pharmacokinetics and radiological response to therapy. By stratifying tumor types, the negative clearance-response relationship was highly significant in NSCLC, and a non-significant trend was seen in RCC. The clearance-response relationship could not be confirmed in melanoma.

Our developed PPK model was comparable to previously described models for different MoAbs and nivolumab. In a model-based meta-analysis for four different MoAbs a robust fit was obtained using a two-compartment model with estimates for systemic clearance and volume of distribution of 0.200 L/day and 3.6 L, respectively, and with a 31% inter-subject variability of clearance [20]. This was similar to recently published parameter estimates of a nivolumab-based two-compartment model described by Bajaj and colleagues (clearance 0.226 L/day; compartmental volume 3.6; 35% inter-subject variability of clearance) [9]. Although the time-varying pharmacokinetics as described by Liu et al. [21] could not be confirmed in our analysis, our time-stationary PPK model worked with sufficient accuracy. Important to note is that we observed that trough levels reached steady state concentrations and remained stable over time in individual patients (as illustrated by Fig. 1b).

Estimated systemic drug clearance according to the PPK model, which is inversely proportional to exposure, was utilized in this study to correlate clearance with response, survival, and occurrence of irAEs. It is important to realize that correlating systemic clearance to treatment outcome using a PPK model may potentially be influenced by those incorporated baseline covariates that are related to the efficacy of immunotherapy. To avoid such confounding, we utilized the initial model to demonstrate the relationship of systemic clearance with clinical outcome, which was further grouped by tumor type. Two patients were excluded from these analyses: a patient with mesothelioma and a patient who was treated with concomitant ipilimumab from the second cycle of nivolumab, at which point he was censored from the analyses.

The differential clearance-response relationship of nivolumab in NSCLC compared to melanoma remains to be elucidated. This may be explained by different tumor intrinsic as well as extrinsic factors, such as tumor immunogenicity and patient immunity (e.g. microbiota, environmental factors) respectively. In addition, positive D-R relationships were found for NSCLC at doses from 0.1 to 3 mg/kg Q2W [10], whereas for melanoma this was only observed at doses from 0.1 to 1 mg/kg Q2W [10]. In this respect it is noteworthy that the ORR of nivolumab in metastatic melanoma is superior compared to metastatic NSCLC [1–3]. In our study, patients with melanoma demonstrated better performance than patients with NSCLC before initiation of nivolumab monotherapy (WHO performance score of 0: 76 and 23%, respectively). Taken together, we cannot exclude that nivolumab may be effective at lower doses for patients with melanoma than for NSCLC, and that patients with NSCLC may be more sensitive for changes in exposure than patients with melanoma. Consequently, nivolumab dosing based on weighted parameters may be relevant to optimize efficacy, particularly for NSCLC patients.

Whether the inverse clearance-response relation for nivolumab in NSCLC indicate a true causal effect on clinical outcome remains to be clarified. The issue was discussed by Badawi in a comment on our previous work showing a positive E-R relationship of nivolumab for NSCLC [13, 26]. Of note, an absent E-R relationship but a strong negative clearance-OS association has been described for pembrolizumab, another PD-1 blocking monoclonal antibody, in solid tumors [27]. The efficacy of pembrolizumab seemed to be independent of absolute doses from 2 to 10 mg/kg Q2W or flat dose 200 mg Q3W. Turner and colleagues argued that this clearance-OS relationship was confounded by the cachectic state of patients, which caused more rapid protein (and thus antibody) turnover and worse survival [27]. Given the circular reasoning in their study design, however, this hypothesis may not have been supported by appropriate evidence [28]. Although we did not find a covariate effect of prior weight loss or the performance score on the PPK model, serum albumin was inversely correlated with nivolumab clearance. Albumin may be considered as a surrogate marker for protein clearance, coinciding with clearance of endogenous immunoglobulins and nivolumab. This may provide supporting evidence to the hypothesis that the exposure-response (or inverse clearance-response) relationship is affected by the metabolic state of patients, although we cannot rule out the possibility of a true causal relationship.

The elimination and recycling mechanisms of nivolumab is expected to exhibit similarities to those of endogenous immunoglobulins with a central role for the neonatal Fc receptor (FcRn) [29]. The FcRn is being widely expressed in human tissue (www.proteinatlas.org), particularly in myeloid cells, and reported to play a key role in the metabolism of albumin in blood [30]. Interestingly, myeloid cells have emerged as major regulators of cancer immune response by presenting tumor antigens to T cells and controlling the activity of cytotoxic cells, where myeloid-derived suppressor cells (MDSCs) suppress T cell responses and are implicated in tumor metastasis [31, 32]. In fact, MDSCs were negatively associated with response to treatment and survival in NSCLC [33, 34]. Monocytic myeloid cells in peripheral blood prior to anti-PD-1 nivolumab and pembrolizumab are associated with inferior PFS and OS [35]. We expect that by further exploring the interaction between the peripheral immune system, e.g. FcRn in the myeloid lineage, and nivolumab pharmacokinetics, understanding of immune checkpoint inhibitor treatment could be significantly enhanced.

## Conclusions

In oncology, pharmacokinetic modeling is widely used to optimize drug dosing. For immune checkpoint inhibitors, approved flat dosing regimens for nivolumab have been solely based on simulations from dose-finding clinical trials. In this prospective real-life patient cohort study, we observed an effect of gender, body surface area, and baseline serum albumin on systemic drug clearance, thereby providing understanding of the high inter-individual variance of clearance. We demonstrate a strong inverse correlation of drug clearance and response in NSCLC, and a similar trend in RCC, but a clearance-response relationship was not observed in melanoma. Considering the recent approval of nivolumab fixed dosing regimens --which was solely based on simulating PK data from clinical studies-- this real-world study suggests that dosing regimens based on patient parameters may be considered to improve efficacy, particularly in NSCLC, and should be prospectively studied.

*Dutch Trial Registry NL6828. Registered 5 April 2016,* *https://www.trialregister.nl/trial/6828* *.*

## Additional file

Additional file 1: **Figure S1.** PPK model. **Figure S2.** Clearance-toxicity analysis. **Figure S3.** Goodness of fit. **Table S1.** Parameter estimates. **Table S2.** Clinical outcome and toxicity. **Table S3.** Patient characteristics by quartiles of drug clearance. Appendix 1 The final and initial models were internally validated using VPC (Figure S3) and a bootstrap procedure. Bootstrap analysis was performed with replacement by randomly selecting patients from the dataset. Syntax of initial PPK model M_i_: (DOCX 588 kb)

## Data Availability

The datasets used and/or analyzed during the current study are available from the corresponding author on reasonable request.

## Abbreviations

BOR: Best overall response
BSA: Body surface area
BSV: Between-subject variability
CKD-EPI: Chronic Kidney Disease Epidemiology Collaboration
CL: Clearance
CR: Complete response
D-R: Dose-response
ELISA: Enzyme-linked immune sorbent assay
E-R: Exposure-response
FcRn: Neonatal Fc receptor
IgG4: Immunoglobulin G4
IQR: Interquartile range
irAE: Immune-related adverse event
LD: Lactate dehydrogenase
MDSC: Myeloid-derived suppressor cell
MoAb: Monoclonal antibody
NCI-CTCAE: National Cancer Institute Common Terminology Criteria for Adverse Events
NSCLC: Non-small cell lung cancer
OFV: Objective function value
OR: Objective response
OS: Overall survival
PD: Progressive disease
PD-1: Programmed death-1
PFS: Progression-free survival
PK: Pharmacokinetics
PPK: Population pharmacokinetics
PR: Partial response
RCC: Renal cell cancer
RECIST: Response Evaluation Criteria in Solid Tumors
SD: Stable disease
