# Supplementary material for: A prospective cohort study on the pharmacokinetics of nivolumab in metastatic non-small cell lung cancer, melanoma, and renal cell cancer patients
Source: J Immunother Cancer. 2019 Jul 19;7:192. doi: 10.1186/s40425-019-0669-y (PMC6642527; doi:10.1186/s40425-019-0669-y)
Supplement: Supplementary file 1 — Figure S1. PPK model. Figure S2. Clearance-toxicity analysis. Figure S3. Goodness of fit. Table S1. Parameter estimates. Table S2. Clinical outcome and toxicity. Table S3. Patient characteristics by quartiles of drug clearance. Appendix 1 The final and initial models were internally validated using VPC (Figure S3) and a bootstrap procedure. Bootstrap analysis was performed with replacement by randomly selecting patients from the dataset. Syntax of initial PPK model Mi: (DOCX 588 kb) [file 40425_2019_669_MOESM1_ESM.docx]

**Additional file 1**

**Figure S1: PPK model**


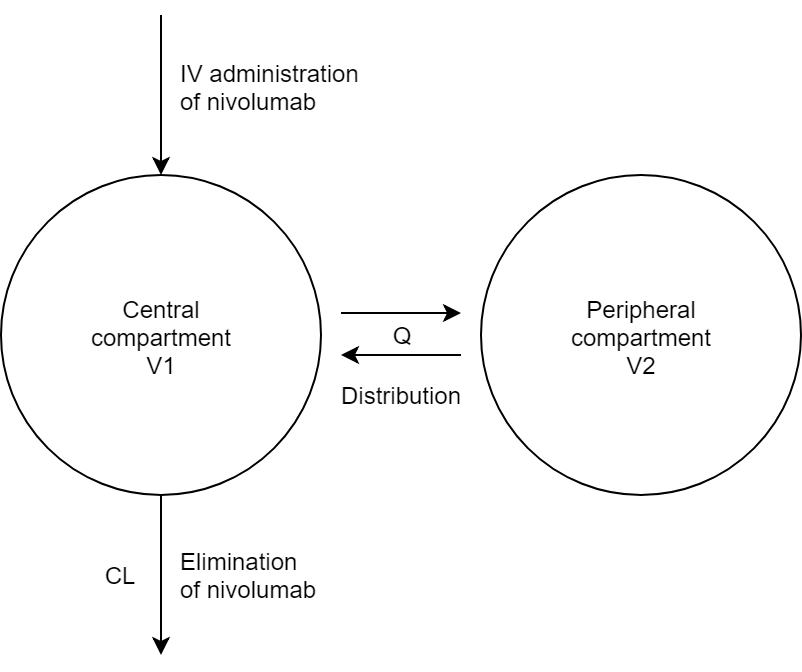


**Figure S1**: Schematic overview of the two-compartmental PPK model of nivolumab.

**Figure S2: Clearance-toxicity analysis**


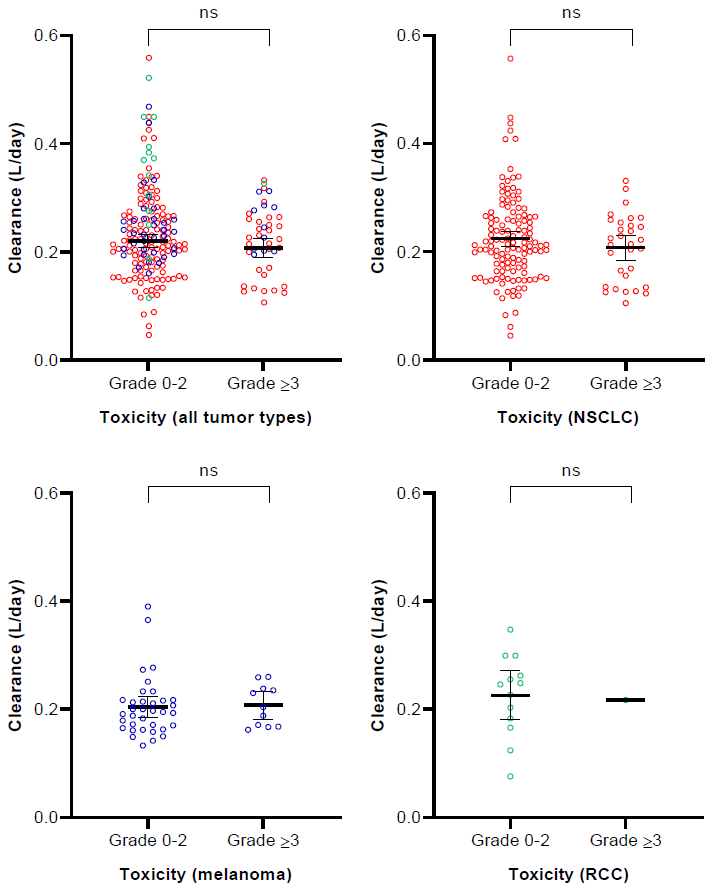


**A B**

**C D**

**Figure S2**: A) Drug clearance (L/day), according to initial model M_i_, grouped by toxicity (irAEs; grade 0-2 vs. grade >3) of A) all patients, B) NSCLC (in red), C) melanoma (in blue) and D) renal cell cancer (RCC; in green) receiving nivolumab monotherapy. Single measurements are represented by open circles. Bars indicate the 95% confidence interval of the mean.

**Figure S3: Goodness of fit**

**
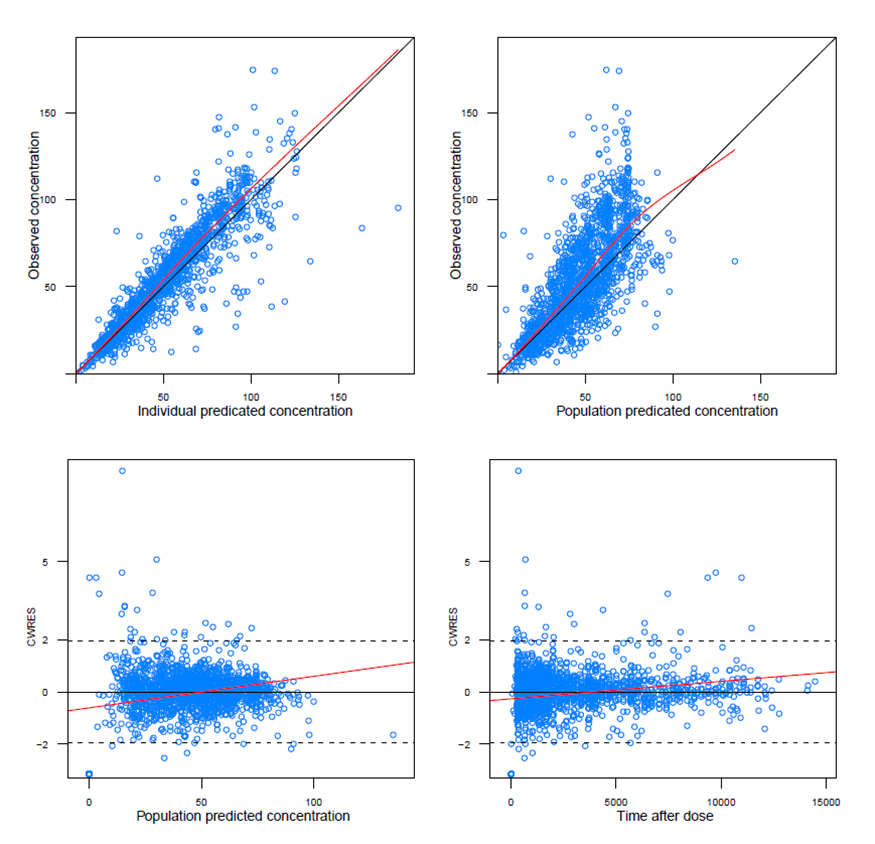
A B
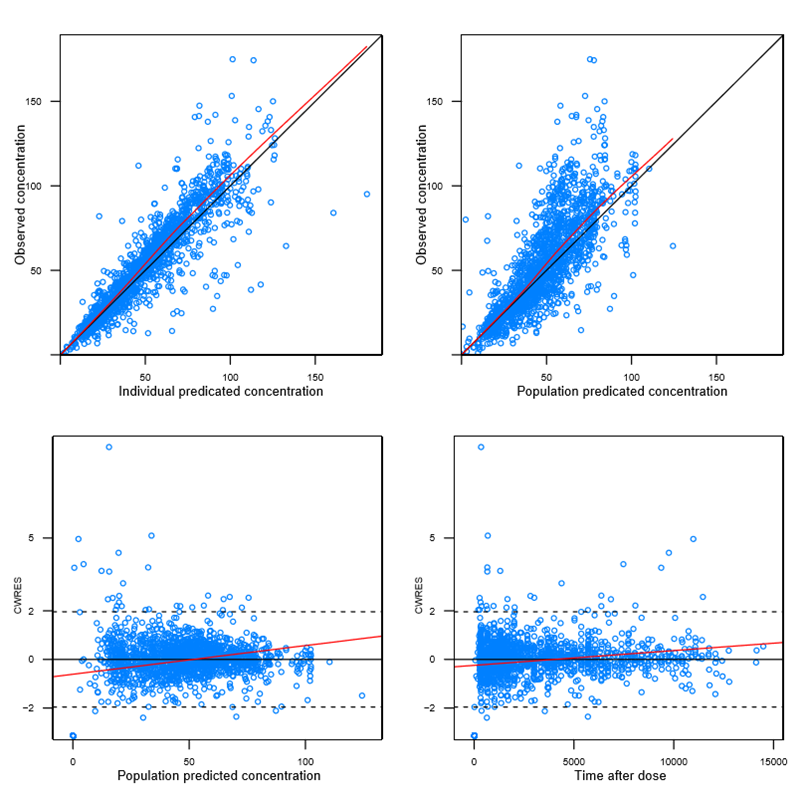
**

**Figure S3**: Standard goodness of fit of the A) initial (Mi) and B) final model (M_f_). Single observations are represented by open circles. Red and black lines mark locally estimated scatterplot smoothing lines, lines of identity (diagonal) or ordinate value of 0 (horizontal), respectively. The figures include observations versus individual predictions (a), observations vs population predictions (b), conditional weighted residuals (CWRES) versus population predictions (c) and CWRES versus time after dose (d).

**Table S1: Parameter estimates**

| Categorical Covariates | Estimates | dOFD |
| --- | --- | --- |
| NSCLC vs. other tumor types | -0.03 | -0.251 |
| Melanoma vs. other tumor types | 0.05 | -0.449 |
| Female vs. Male | -0.26 | -29.685 |
| WHO=0 vs. Rest (including unknowns) | 0.08 | -1.682 |
| Weight Loss Present vs. Rest (including unknowns) | 0.09 | -1.406 |

| Continuous Covariates | Estimates | dOFD |
| --- | --- | --- |
| Age (yr) | 0.12 | -0.604 |
| Body Weight (kg) | 0.60 | -18.716 |
| Body Surface Area (m^2^) | 1.22 | -25.519 |
| Tumor Burden 3D (cm^3^) | 0.09 | -2.252 |
| Creatinine (μmol/L) | -0.01 | -0.003 |
| CKD (mL/min) | 0.01 | -0.307 |
| Total Protein (g/L) | 0.25 | -0.300 |
| Albumine (g/L) | -1.31 | -18.989 |
| LD (U/L) | 0.0004 | - |
| Leucocytes (10^9^ cells/L) | 0.07 | -0.648 |

**Table S1**: Categorical and continuous covariates shown as estimates and difference in objective function value according to initial model Mi (dOFD). Abbreviations: CKD-EPI renal clearance (CKD), lactate dehydrogenase (LD).

**Table S2: Clinical outcome and toxicity**

|  | Total n(%) | NSCLC n(%) | Mel n(%) | RCC n(%) |
| --- | --- | --- | --- | --- |
| BOR (RECIST v1.1) |  |  |  |  |
| PR/CR | 53 (24.2) | 28 (17.7) | 22 (46.8) | 3 (21.4) |
| SD | 57 (26.0) | 47 (29.7) | 7 (14.9) | 3 (21.4) |
| PD | 97 (44.3) | 73 (46.2) | 18 (38.3) | 6 (42.9) |
| Non-evaluable | 12 (5.5) | 10 (6.3) | - | 2 (14.3) |
| IrAEs (CTCAE 4.03) | | |  |  |
| Grade 0-2 | 175 (79.9) | 127 (80.4) | 35 (74.5) | 13 (92.9) |
| Grade >3 | 43 (19.6) | 31 (19.6) | 11 (23.4) | 1 (7.1) |
| Unknown | 1 (0.5) | - | 1 (2.1) | - |

**Table S2**: Distributions of best overall response (BOR) by RECIST v1.1 and irAEs by CTCAE v4.03 for all patients receiving nivolumab monotherapy and grouped by tumor type. Abbreviations: melanoma (Mel), renal cell carcinoma (RCC).

**Table S3: Patient characteristics by quartiles of drug clearance**

|  | Q1 | Q2 | Q3 | Q4 |
| --- | --- | --- | --- | --- |
| Tumor type *n (% within quartile)* |  |  |  |  |
| NSCLC | *n=39* | *n=40* | *n=40* | *n=39* |
| Adenocarcinoma | 23 (59) | 30 (75) | 23 (57.5) | 20 (51) |
| Squamous cell carcinoma | 11 (28) | 7 (17.5) | 10 (25) | 14 (36) |
| Unknown | 5 (13) | 3 (7.5) | 7 (17.5) | 5 (13) |
| WHO performance score *n (% within quartile)* |  |  |  |  |
| 0 | 5 (13) | 8 (20) | 7 (17.5) | 5 (13) |
| 1 | 25 (64) | 16 (40) | 19 (47.5) | 23 (59) |
| 2 | - | 1 (2.5) | - | 1 (2.5) |
| Unknown | 9 (23) | 15 (37.5) | 14 (35) | 10 (25.5) |
| Weight loss prior to start therapy *n (% within quartile)* |  |  |  |  |
| Yes | 12 (31) | 12 (30) | 5 (12.5) | 7 (18) |
| No | 17 (43.5) | 19 (47.5) | 21 (52.5) | 23 (59) |
| Unknown | 10 (25.5) | 9 (22.5) | 14 (35) | 9 (23) |
| Gender *n (% within quartile)* |  |  |  |  |
| Male | 11 (28) | 23 (57.5) | 29 (72.5) | 35 (90) |
| Female | 28 (72) | 17 (42.5) | 11 (27.5) | 4 (10) |
| Body Surface Area (m^2^) *median (IQR)* | 1.81 (1.55-1.97) | 1.85 (1.76-2.06) | 1.95 (1.84-2.10) | 2.00 (1.91-2.13) |
| Albumin (g/L) *median (IQR)* | 42 (41-44) | 43 (40-45) | 42 (40-45) | 39 (34-43) |

**Table S3**: Patient characteristics including important patient parameters (gender, BSA and serum albumin) grouped by quartile of drug clearance (Q1-Q4), which was utilized for the clearance-survival analysis. The percentage within the quartiles is shown, leaving out the unknown parameters. Abbreviations: Q1-4 (quartile 1-4), IQR (inter-quartile range).

**Appendix 1**

The final and initial models were internally validated using VPC **(Suppl. Figure** **3)** and a bootstrap procedure. Bootstrap analysis was performed with replacement by randomly selecting patients from the dataset.

Syntax of initial PPK model M_i_:

$PROBLEM PK

$INPUT DROP ID CYCLE TIME DV AMT RATE MDV EVID CMT DAY WEEK TAD RESPONS GRTT

$DATA FINAL_PK_MULTOMAB_NSCLC_RCC_MEL.csv IGNORE=#;concentration mg/L

$SUBROUTINES ADVAN3 TRANS4

$PK

CL = THETA(1)*EXP(ETA(1))

V1 = THETA(2)

V2 = THETA(2)

Q= THETA(3)

S1 = V1

$ERROR

TY=F

IF(F.LT.0.001) TY=0.001

IPRED = TY

Y=IPRED*(1+ERR(1))

IRES = DV-IPRED

IWRES = IRES/IPRED

$THETA

(0, 0.01) ; CL

(0, 5) ; V1

0.02 FIX; Q

$OMEGA

(0.1) ; IIV CL

$SIGMA

0.05 ; prop paz

$EST METHOD=1 INTER MAXEVAL=2000 NOABORT SIG=3 PRINT=1 POSTHOC

$COV

Syntax of final PPK model M_f_:

$PROBLEM PK

$INPUT DROP ID CYCLE TIME DV AMT RATE MDV EVID CMT DAY WEEK TAD RESPONS

GRTT SEX RACE DISEASE AGE WHO LGT WGT BSA TWOD THREED CREAT CKD TPRO ALB

LD LEU GRAN EOS NEUIMM NEUMAT BC MON DC TC CDF CDE CDFP CDEP WLB WLP

$DATA FINAL_PK_MULTOMAB_NSCLC_RCC_MEL.csv IGNORE=#;concentration mg/L

$SUBROUTINES ADVAN3 TRANS4

$PK

;CLSEX-DEFINITION START

IF(SEX.EQ.0) CLSEX = 1 ; Most common

IF(SEX.EQ.1) CLSEX = 1 + THETA(4)

;CLSEX-DEFINITION END

;CLBSA-DEFINITION START

IF(BSA.EQ.999) THEN

CLBSA=1

ELSE

CLBSA=(BSA/1.9)**THETA(5)

ENDIF

;CLBSA-DEFINITION END

;CLALB-DEFINITION START

IF(ALB.EQ.999) THEN

CLALB=1

ELSE

CLALB=(ALB/42)**THETA(6)

ENDIF

;CLALB-DEFINITION END

;CL-RELATION START

CLCOV=CLSEX*CLBSA*CLALB

;CL-RELATION END

TVCL=THETA(1)

CL = TVCL*CLCOV*EXP(ETA(1))

V1 = THETA(2)

V2 = THETA(2)

Q= THETA(3)

S1 = V1

$ERROR

TY=F

IF(F.LT.0.001) TY=0.001

IPRED = TY

Y=IPRED*(1+ERR(1))

IRES = DV-IPRED

IWRES = IRES/IPRED

$THETA

0.0085 ; CL

3.45 ; V1

0.02 FIX; Q

(-3,-.261) ; CLSEX

(-3,1.22) ; CLBSA

(-3,-1.31) ; CLALB

$OMEGA

(0.1) ; IIV CL

$SIGMA

0.05 ; prop paz

$EST METHOD=1 INTER MAXEVAL=2000 NOABORT SIG=3 PRINT=1 POSTHOC

$COV
